# Supplementary material for: Preclinical characterization of immune responses induced by a candidate gonococcal native outer membrane vesicle vaccine
Source: mSphere. 2026 Jun 22;11(7):e00174-26. doi: 10.1128/msphere.00174-26 (PMC13410757; doi:10.1128/msphere.00174-26)
Supplement: Supplemental Figures — Fig. S1 to S4. [file msphere.00174-26-s0001.docx]

**Preclinical characterization of immune responses induced by a candidate gonococcal native outer membrane vesicle**

**Supplemental Information**

**Figure S1:** **Experimental design to evaluate the immunogenicity in mice of GonoVac in a dose escalation study with and without Al(OH)_3_.** Groups of six 6–8-week-old BALB/c mice were immunized intramuscularly with doses of GonoVac ranging from 5 µg to 0.15 µg (total protein content) produced in a bioreactor or 5 µg produced in shake flasks, formulated with or without Al(OH)₃. The 4CMenB control group received 5 µg of the dOMV protein equivalent. Naïve mice served as a non-immunized control group, while the Al(OH)₃ negative control group received adjuvant only. Each group received 3 immunizations of the assigned vaccine at 3-week intervals (week 0, week 3, week 6), and blood samples were collected 3 weeks post each dose at weeks 3, 6 and 9. Spleens and vaginal lavages were collected post-mortem at week 9.

**
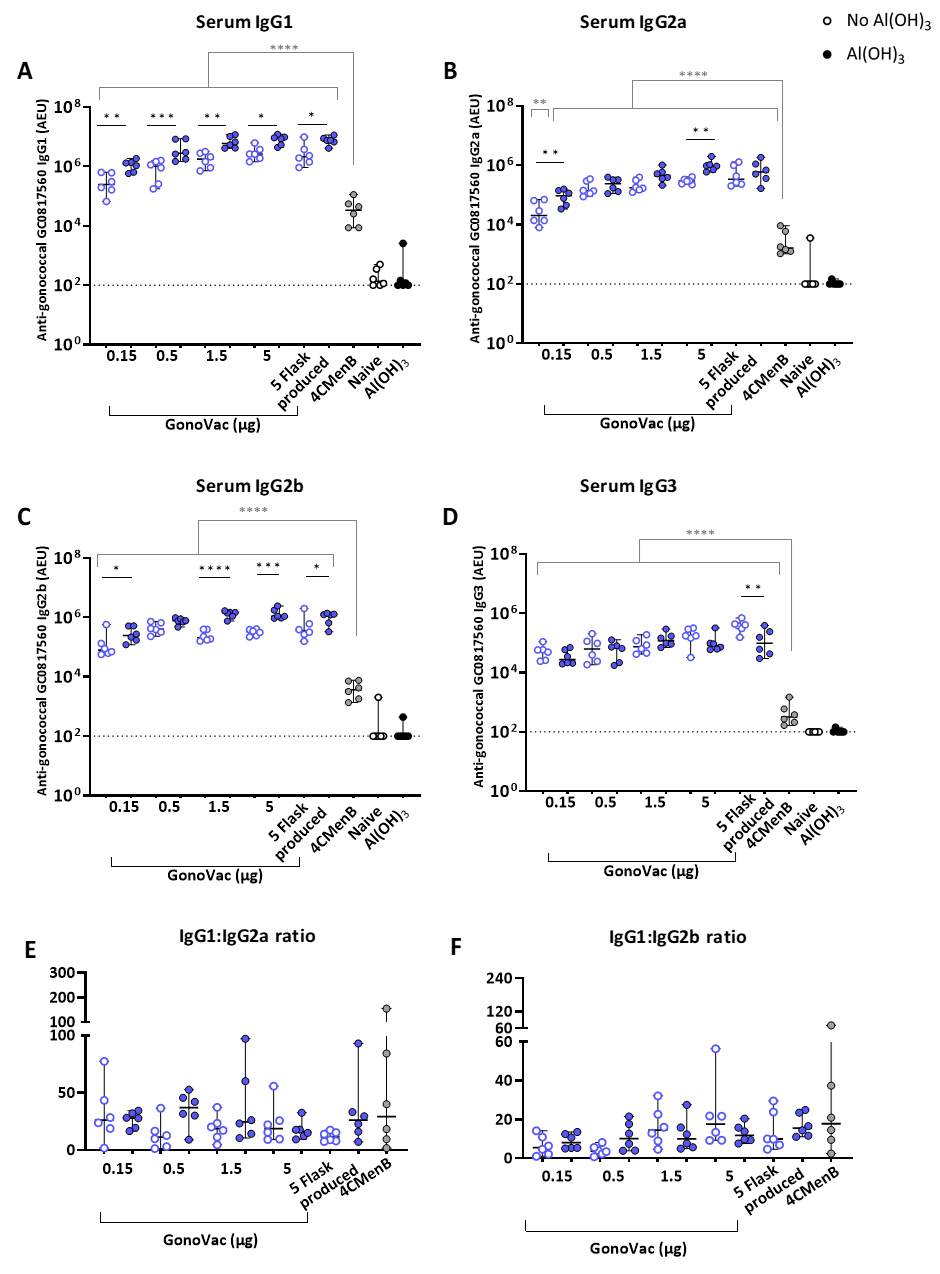
**

**Figure S2: Serum anti-gonococcal IgG subclass responses following a dose escalation of GonoVac with and without Al(OH)₃ in mice.** 6-8 week-old BALB/c mice were immunized with escalating doses (0.15-5 µg) of GonoVac produced in a bioreactor or 5 µg produced in shake flasks, formulated with and without Al(OH)₃, 5 µg of 4CMenB or Al(OH)_3_ alone as control. Naïve mice were not immunized. Mice received 3 doses at 3-week intervals (days 0, 21, and 42). Anti-gonococcal IgG subclasses (A) IgG1, (B) IgG2a, (C) IgG2b and (D) IgG3 were determined in serum samples collected after 3 immunizations by ELISA using lysate of the GC_0817560 strain as antigen. All the results are expressed in Arbitrary ELISA Units (AEU). The horizontal dashed line represents the limit of quantification (LOQ), determined as the AEU of the lowest point of the standard curve adjusted by the minimum dilution factor applied to the samples. Serum IgG1 and IgG2a titers were used to calculate (E) IgG1:IgG2a and (F) IgG1:IgG2b providing insight into the type of immune response generated: a higher ratio suggests a predominant Th2-type response, while a lower ratio indicates a stronger Th1-type response. Individual mice are represented by circles (median ± 95% CI; n=6). Statistical test: 2-way ANOVA with Šídák's correction for multiple comparisons; grey stars indicate the significance compared to 4CMenB. P values: * p<0.05, ** p<0.01, *** p<0.001, **** p<0.0001.


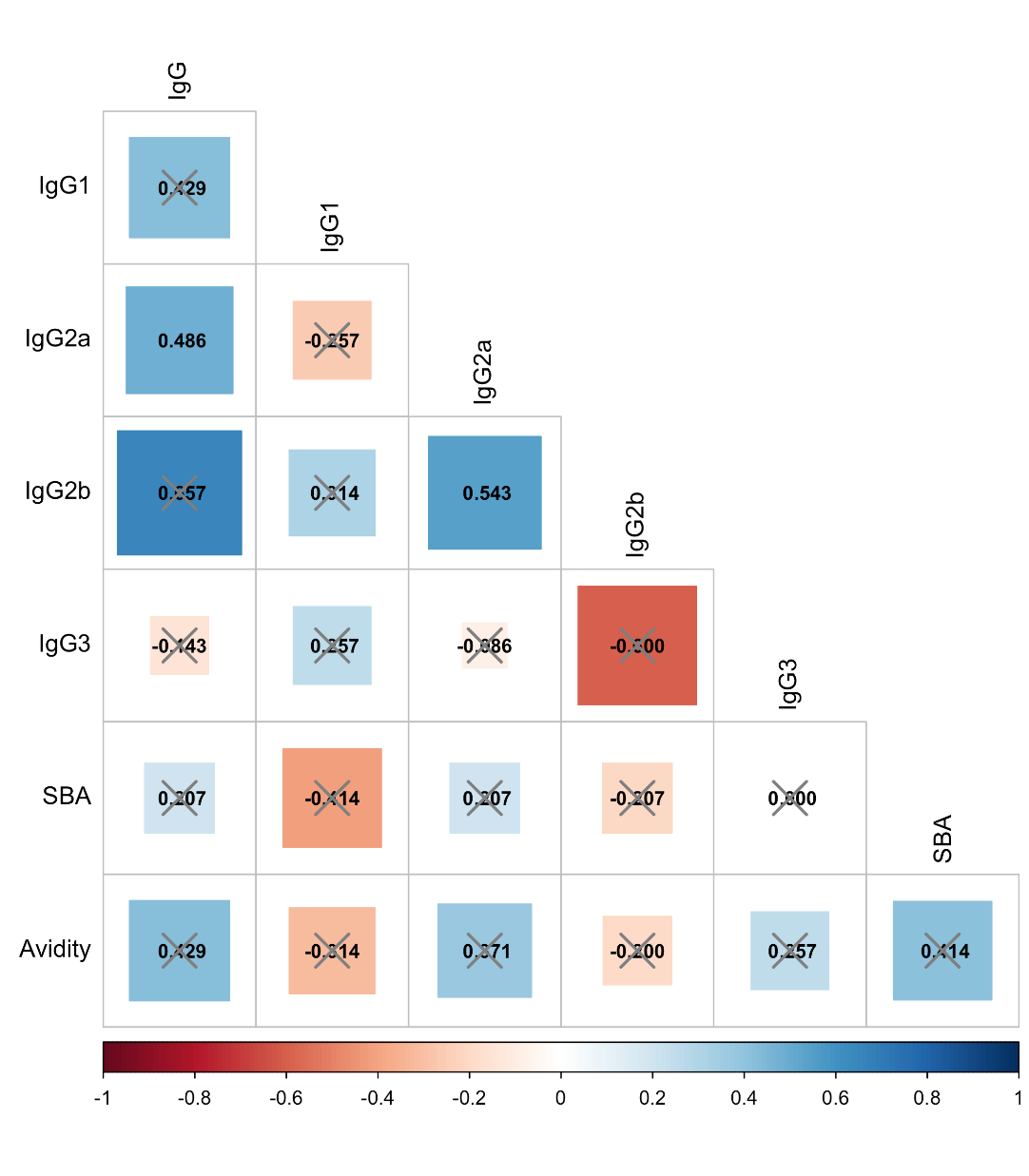


**Figure S3: Correlation matrix between 4CMenB-induced serum anti-gonococcal IgG, IgG subclasses, serum bactericidal titers and IgG avidity.** The matrix was generated in R using the corrplot package and is based on Spearman’s rank correlation coefficients calculated from data obtained from mice immunised with three doses of 4CMenB (5 µg). Pairwise correlations that were not statistically significant at p < 0.05 are marked with a cross.

**
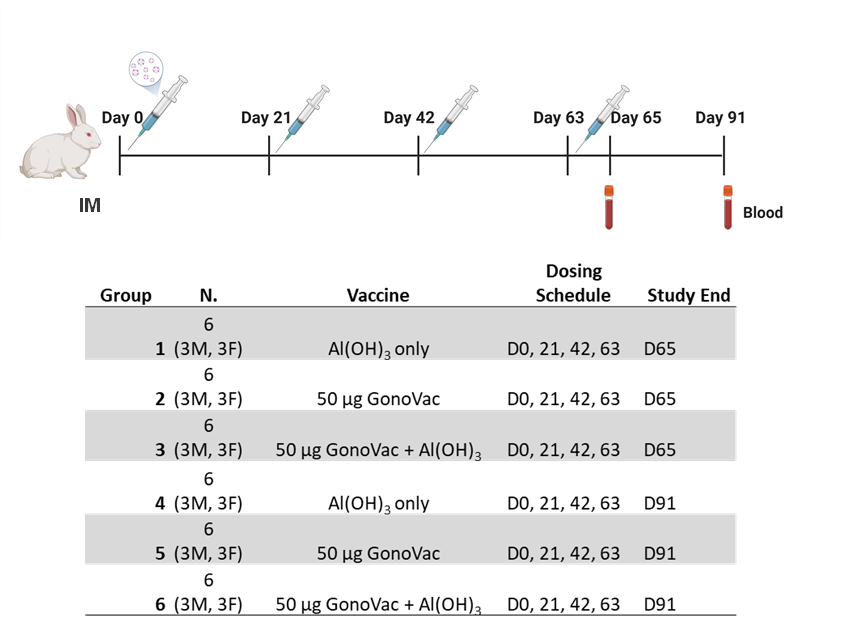
**

**Figure S4: Experimental design to evaluate the immunogenicity in rabbits of GonoVac with and without Al(OH)_3_.** Groups of six white New Zealand rabbits (three male and three female per group) received 4 doses of 50 µg GonoVac intramuscularly with and without Al(OH)_3_ or with Al(OH)_3_ alone. Two groups were included for each condition: one group was sacrificed 65 days post 1st dose and the other group 95 days following the 1st immunization.
